# Key-Frame-Aware Hierarchical Learning for Robust Gait Recognition

**DOI:** 10.3390/jimaging11110402

**Published:** 2025-11-10

**Authors:** Ke Wang, Hua Huo

**Affiliations:** 1 College of Information Engineering, Henan University of Science and Technology, Luoyang 471023, China; wklutwk@163.com; 2 Henan Provincial Engineering Research Center for Artificial Intelligence Technology and Application in Distance Education, Henan Open University, Zhengzhou 450046, China

**Keywords:** gait recognition, key-frames, hierarchical spatio-temporal representation, frame-level feature re-segmentation

## Abstract

Gait recognition in unconstrained environments is severely hampered by variations in view, clothing, and carrying conditions. To address this, we introduce HierarchGait, a key-frame-aware hierarchical learning framework. Our approach uniquely integrates three complementary modules: a TemplateBlock-based Motion Extraction (TBME) for coarse-to-fine anatomical feature learning, a Sequence-Level Spatio-temporal Feature Aggregator (SSFA) to identify and prioritize discriminative key-frames, and a Frame-level Feature Re-segmentation Extractor (FFRE) to capture fine-grained motion details. This synergistic design yields a robust and comprehensive gait representation. We demonstrate the superiority of our method through extensive experiments. On the highly challenging CASIA-B dataset, HierarchGait achieves new state-of-the-art average Rank-1 accuracies of 98.1% under Normal (NM), 95.9% under Bag (BG), and 87.5% under Coat (CL) conditions. Furthermore, on the large-scale OU-MVLP dataset, our model attains a 91.5% average accuracy. These results validate the significant advantage of explicitly modeling anatomical hierarchies and temporal key-moments for robust gait recognition.

## 1. Introduction

Human gait, the characteristic manner of walking, serves as a viable biometric modality for identification. Its primary advantage lies in its potential for non-cooperative recognition at a distance, unlike biometrics requiring close proximity or subject interaction, such as fingerprints or iris scans [1]. This unique capability has garnered significant interest across diverse application domains. In forensic science and surveillance, gait analysis can aid in identifying individuals captured in video footage, even when other features are obscured [2,3]. Sports science utilizes gait analysis for optimizing athletic performance, assessing injury risks, and monitoring rehabilitation progress [4,5]. Furthermore, in intelligent transportation and autonomous systems, robust pedestrian gait recognition contributes to safer navigation and more accurate human behavior prediction [6,7].

Despite this potential, the practical deployment of gait recognition systems is hindered by substantial challenges inherent in capturing gait under unconstrained conditions. Variations in viewing angles significantly alter the perceived silhouette shape and dynamics [7]. Partial occlusions caused by environmental objects or other people can obscure critical body parts [8]. Moreover, intra-subject variations such as changes in clothing [9], carrying conditions (e.g., bags, backpacks), or walking speed introduce considerable variability into the observed gait pattern. Addressing these factors is paramount for developing reliable and widely applicable gait recognition technology.

Consequently, the development of robust feature representations capable of handling these variations remains an active research area. Early methods often relied on holistic representations, such as Gait Energy Images (GEIs) [10], which average silhouettes over a gait cycle, providing a compact spatial template but sacrificing temporal details. To better capture motion dynamics and fine-grained spatial information, sequence-based approaches operating directly on silhouettes [11] or derived representations have become prevalent. These methods often leverage deep learning, particularly Convolutional Neural Networks (CNNs), to learn discriminative spatio-temporal features [10,12]. Pre-processing typically includes silhouette extraction and alignment [13] to normalize the input sequences spatially. Building upon sequence analysis, part-based methods [14,15] emerged to enhance robustness against local variations. By dividing the human body silhouette or intermediate feature maps into distinct regions (commonly horizontal strips) and processing them somewhat independently, these methods aim to isolate the effects of variations, such as different trousers affecting only the lower body strips or a carried bag primarily impacting upper body features.

**Figure 1:**
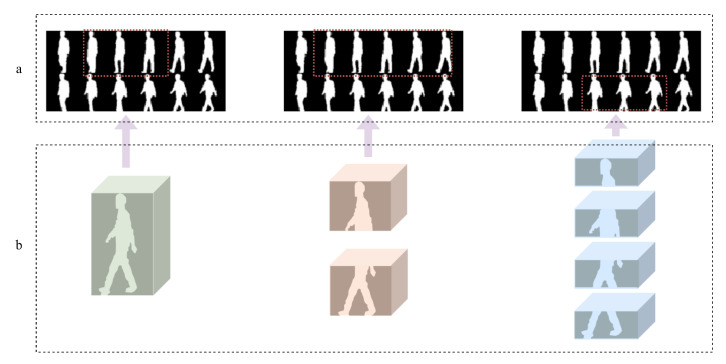
Conceptual illustration of the core motivations for our framework. The gait frames within the red dashed box represent the key-frames. (**a**) Key-Frame Importance: In a typical gait sequence, certain frames (highlighted in red dashed box) contain more discriminative spatio-temporal information than others. Our SSFA module is designed to identify and emphasize these key-frames. (**b**) Hierarchical Motion Dependencies: Human motion has an inherent hierarchy. Our TBME module is designed to capture these dependencies by analyzing motion from a coarse-to-fine perspective, moving from the whole body to upper/lower body and finally to individual limb segments.

However, existing part-based approaches often overlook two critical aspects, limiting their potential for capturing the full complexity of human gait:

Hierarchical Motion Dependencies: First, they often neglect the inherent hierarchical structure of human motion during gait. As illustrated conceptually in Figure 1b, walking involves complex, coordinated movements across different body levels (e.g., the motion of the calf is directly influenced by the thigh, and both contribute to the overall leg swing dynamics relative to the torso). Standard part-based methods, typically employing simple horizontal slicing, fail to explicitly model these multi-level kinematic dependencies. By treating predefined spatial regions largely independently or with only local connections, they risk overlooking the crucial inter-segment relationships and coordinated dynamics that define an individuals’ unique walking pattern. Capturing this hierarchy is vital for a more biomechanically plausible and potentially more discriminative feature representation.

Key-Frame Importance: Second, they frequently overlook the varying importance of different temporal phases within a gait sequence. As depicted in Figure 1a, certain ’key-frames’ or temporal segments often encapsulate the most discriminative spatio-temporal information—moments capturing characteristic poses like maximum limb extension, heel strike, or specific torso postures during the swing or stance phase. Existing methods commonly aggregate features across time either uniformly (e.g., temporal pooling) or without an explicit mechanism designed to identify and emphasize these information-rich moments. This uniform temporal treatment can dilute the contribution of the most characteristic postures and transitions by averaging them with less informative or potentially noisy frames, thereby hindering the model’s ability to focus on the most salient temporal dynamics for robust recognition.

To overcome these limitations, we argue that an effective gait recognition system should explicitly model the hierarchical nature of body motion and emphasize the discriminative information present in key-frames. To this end, this paper introduces a novel framework for key-frame-aware hierarchical learning. Our primary contributions are delineated as follows:

- Novel Key-Frame-Aware Hierarchical Learning Framework: We propose a deep learning framework that integrates hierarchical spatial decomposition with key-frame-aware temporal aggregation. Central to this is the Template Block-based Motion Extraction (TBME) module, which uses non-shared 3D convolutions tailored to predefined anatomical levels (e.g., whole body, upper/lower body, limbs), enabling a structured coarse-to-fine feature extraction process that captures intrinsic kinematic dependencies.
- Key-Frame Focused Temporal Aggregation (SSFA): Recognizing that not all frames contribute equally to identification, we introduce the Sequence-Level Spatio-temporal Feature Aggregator (SSFA). This module adaptively identifies and aggregates features from the most discriminative moments (key-frames) of the gait cycle, creating a compact and salient gait signature that is robust to temporal noise.
- Fine-Grained Frame-Level Feature Refinement (FFRE): To capture intricate local details missed by coarser representations, we introduce the Frame-level Feature Re-segmentation Extractor (FFRE). This module enhances the spatial resolution of features by dynamically re-segmenting feature maps of specific body parts within each frame, allowing the model to learn localized motion characteristics (e.g., foot rotation) with greater precision.
- State-of-the-Art Performance through Synergistic Integration: The synergistic integration of TBME, SSFA, and FFRE results in a powerful and comprehensive gait representation. We demonstrate through extensive experiments on the challenging CASIA-B and large-scale OU-MVLP datasets that our method achieves state-of-the-art performance, validating the effectiveness of jointly modeling anatomical structure and temporal significance. Unlike prior methods that typically focus on either hierarchical features or temporal modeling in isolation, our framework’s novelty lies in their synergistic integration, creating a more holistic representation that captures local, global, structural, and temporal dynamics simultaneously.

## 2. Related Work

### 2.1. Gait Recognition

Currently, deep learning-based gait recognition techniques are emerging as a focal point of research, attributed to their remarkable capabilities in feature learning and representation. These methodologies primarily fall into two distinct categories: model-based approaches [16] and appearance-based techniques [12].

Firstly, we delve into model-driven gait recognition methodologies. The essence of these methods involves extracting the structural intricacies and locomotive patterns of the human body from gait videos, leveraging pose estimation techniques [17]. By harnessing the capabilities of deep learning networks, researchers are capable of precisely pinpointing the joint locations of the human body, thereby facilitating the construction of three-dimensional human skeleton models [17]. These models offer an intuitive visualization of the posture variations of the human body during locomotion, affording a wealth of information pertinent to gait recognition.

However, model-based approaches pose certain challenges that need to be addressed. Firstly, these methods generally necessitate high-quality video inputs, and any degradation in video resolution or the presence of occlusion factors can potentially compromise the accuracy of pose estimation [18]. Secondly, the intricate process of 3D modeling and computation demands significant computational resources, thereby limiting its widespread application in real-world settings to a certain extent.

In comparison, appearance-based gait recognition methods exhibit greater flexibility and adaptability. These methods facilitate the direct extraction of features from raw images or silhouette sequences of gait videos, thereby eliminating the necessity for intricate 3D modeling. Critical gait-related features, including gait period, step length, swing angle, and other pertinent attributes, can be automatically captured through the utilization of deep learning networks [19]. These features not only demonstrate resilience against variations in viewing angle and appearance, but they also effectively capture the unique walking features of individuals [20].

In recent years, notable advancements have been witnessed in the realm of gait recognition methods that rely on visual appearance. Experts in the field have harnessed deep learning methodologies, particularly convolutional neural networks (CNN) [21], for the purpose of extracting and classifying gait-related features from videos. By meticulously designing effective network architectures and fine-tuning algorithms, precise extraction and efficient classification of gait features have been successfully achieved [22]. Furthermore, several studies have explored the integration of additional biometric information, such as gait sound and gait pressure, in order to further enhance the accuracy and dependability of gait recognition systems [23].

Our proposed method falls within the realm of appearance-based gait recognition methodologies. It employs silhouette sequences as its primary input data, leveraging deep learning algorithms to extract pertinent information that accurately captures gait features. This methodology effectively addresses the inherent challenges posed by low-resolution videos, while also demonstrating exceptional flexibility and practical utility in real-world applications.

### 2.2. Hierarchical Model

Hierarchical feature representation holds a progressively pivotal position in computer vision tasks, particularly when confronted with intricate and layered visual data [15]. Within the realm of person re-identification (ReID), hierarchical techniques extract and integrate features across diverse levels, from local descriptors to global appearance [24,25,26].

In gait recognition, hierarchical feature representation has garnered attention for integrating features across multiple granularities. GaitPart [27] integrates part-level and sequence-level features for a part-independent spatio-temporal representation. CSTL [28] adaptively incorporates multi-scale temporal features for sequence modeling. 3D Local [29] uses localization operations to pinpoint 3D volumes of body parts for fine-grained feature extraction. GaitGL [30] considers both whole-body and part-based information for discriminative learning. STAR [31] employs a Multi-branch Diverse Region Feature Generator and a Spatio-Temporal Augmentation Interactor to capture complex spatio-temporal features across different spatial regions. FATN [32] introduces a Free-Area Transformer to optimize region ratios for efficiency, alongside networks focusing on feature-based and deformation-based recognition. More recently, GaitASMS [33] utilizes adaptive structured spatial mapping and multi-dimensional temporal integration, showing strong performance, particularly in complex scenarios.

However, despite these advancements, many methods still underutilize fine-grained features related to hierarchical motion dependencies or the specific importance of key-frames within the sequence. To address this, our proposed method employs a structured, multi-level hierarchical approach (TBME) combined with fine-grained spatial refinement (FFRE) and key-frame temporal aggregation (SSFA).

To better contextualize our work, Table 1 summarizes several state-of-the-art methods and their core techniques.

### 2.3. Temporal Model

Temporal information is crucial in gait recognition, reflecting rhythmic changes during locomotion. Traditional methods often condense sequences (e.g., GEI [35], GENI [36]) or treat frames as unordered sets (e.g., GaitSet [34], GCEM [37]), potentially losing critical local motion details.

Recent works increasingly focus on explicitly modeling temporal information using spatio-temporal algorithms. 2D and 3D convolutions are widely used [22,38]. For instance, MT3D [38] employs multi-scale 3D convolutions with enhanced pooling. LSTM networks capture long-term dependencies [39], sometimes applied segment-wise with attention. Advanced methods like GaitASMS [33] also incorporate sophisticated multi-scale temporal integration strategies alongside spatial modeling.

While these approaches improve temporal modeling, accurately capturing subtle variations and the discriminative importance of specific moments (key-frames) remains challenging. Our approach differs by employing the SSFA module, which uses a multi-scale spatio-temporal aggregation strategy focused explicitly on identifying and leveraging key-frames, complemented by the hierarchical structure learned via TBME.

In addition to CNN-based hierarchical models, recent years have seen the rise of Transformer-based architectures in gait recognition. Models like GaitFormer [40] and TransGait [41] leverage self-attention mechanisms to capture long-range dependencies in the temporal domain, offering an alternative to recurrent or 3D convolutional approaches. These methods have shown promise in capturing global temporal context. Concurrently, there is a growing focus on developing lightweight models for efficient deployment on edge devices. Research in this area, such as MobileGait [42], explores techniques like network pruning, quantization, and knowledge distillation to create models with a smaller computational footprint, though often with a trade-off in accuracy. Our work differs from these by focusing on a structured, explicit hierarchical decomposition with CNNs, which can be more directly guided by biomechanical priors, while acknowledging the need for future work in model compression for edge deployment. While Transformers excel at capturing long-range global dependencies, our approach deliberately concentrates on a structured, biomechanically-inspired hierarchy, which can offer advantages in feature interpretability and robustness when salient, local motion cues are critical.

In summary, while prior work has significantly advanced appearance-based methods (Section 2.1), explored various hierarchical representations (Section 2.2), and developed sophisticated temporal modeling techniques (Section 2.3), there remains a gap in effectively integrating these aspects within a single framework. Specifically, existing methods often struggle to simultaneously capture intrinsic hierarchical motion dependencies across different body part levels, fine-grained spatial details within individual frames, and the varying temporal significance of frames, particularly key moments in the gait cycle. Our proposed framework, through the synergistic combination of key-frame temporal aggregation (SSFA), hierarchical motion modeling (TBME), and fine-grained spatial refinement (FFRE), directly addresses these limitations, aiming for a more comprehensive and discriminative gait representation.

## 3. Proposed Method

**Figure 2:**
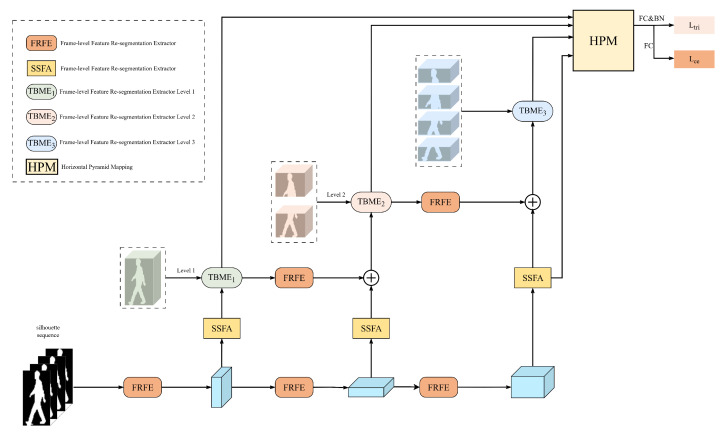
The overall architecture of our proposed HierarchGait framework. The dotted line boxes provide the full names for the module abbreviations. A silhouette sequence is first processed by the FFRE module for fine-grained frame-level feature extraction. The resulting feature sequence is then fed into the SSFA for key-frame temporal aggregation and the TBME for hierarchical motion decomposition. Finally, features from all paths are fused by the HPM module to produce the final gait representation for loss calculation.

As depicted in Figure 2, the architecture of our proposed framework provides a comprehensive description of our approach to processing gait sequences. Commencing with the preprocessing stage, the entire process culminates in the final gait recognition, with the objective of extracting meaningful features from the input gait sequences to facilitate precise human gait recognition.

During the preprocessing phase, the input gait sequence is conceptually partitioned based on a hierarchical model of gait motion (e.g., three levels). Each level involves segmenting the silhouette or feature map horizontally (e.g., into 1, 2, and 4 parts for levels 1, 2, and 3 respectively). These parts often align with anatomical structures (e.g., head/neck, lower/upper leg). This segmentation guides the hierarchical feature extraction process detailed below.

### 3.1. Pipeline Overview

Our proposed pipeline processes gait silhouette sequences to extract a final discriminative feature representation.

First, the input gait sequence, comprising multiple frames $\left\{f_{i},i=1,\ldots ,T\right\}$, is processed frame-by-frame. We employ the Frame-level Feature Re-segmentation Extractor (FFRE), a specialized convolutional network module detailed in Section 3.2, to extract fine-grained spatial features $F_{i}$ for each frame $f_{i}$:

(1) $$F_{i}=\mathrm{FFRE}\left(f_{i}\right)$$

This results in a sequence of frame-level feature maps $S_{F}=\left\{F_{i},i=1,\ldots ,t\right\}$, where each $F_{i}$ is typically a 3D tensor (Channel × Height × Width).

Next, the sequence of frame features $S_{F}$ is fed into the Sequence-level Spatio-temporal Feature Aggregator (SSFA) module (described in Section 3.3). The SSFA module aggregates temporal information across the sequence, focusing on extracting features from discriminative key-frames. It outputs a sequence-level feature representation, denoted as $S_{L}$:

(2) $$S_{L}=\mathrm{SSFA}\left(S_{F}\right)$$

This feature $S_{L}$ is designed to capture essential spatio-temporal motion patterns of the gait cycle.

Subsequently, the sequence-level feature $S_{L}$ enters the core hierarchical processing stage. We employ the Template Block-based Motion Extractor (TBME) module (detailed in Section 3.4). Based on a predefined hierarchy (e.g., *L* levels, with $k_{l}$ parts at level *l*), TBME processes $S_{L}$ to extract multi-granularity motion features specific to different body part levels. For a hierarchy with *L* levels and part sets $m=\{m^{\left(l\right)}|l=1\ldots L\}$, TBME generates hierarchical feature representations $\left\{M^{\left(l\right)}|l=1,\ldots ,L\right\}$. This module uses independent operations (e.g., 3D convolutions) for different hierarchical parts to capture distinct motion characteristics.

To further enhance feature discriminability, these extracted hierarchical gait motion features $M^{\left(l\right)}$ are then passed through another instance of the FFRE module. This step refines the spatio-temporal features at each hierarchical level, producing refined feature maps $\left\{M^{\left(l\right)}|l=1,\ldots ,L\right\}$.

Finally, the parallel extraction of hierarchical spatial features (via TBME and FFRE) and global temporal features (via SSFA), these diverse feature streams converge into a final module inspired by Horizontal Pyramid Matching (HPM) [43]. This module’s role is to fuse these multi-level spatial and temporal cues, creating a unified and comprehensive gait representation. Finally, this representation is passed to separate output heads to compute the Triplet $L_{\mathrm{tri}}$ and Cross-Entropy $L_{\mathrm{ce}}$ losses for joint optimization.

To further clarify the process, Algorithm 1 summarizes the computational pipeline of HierarchGait.
**Algorithm 1** HierarchGait Computational Pipeline  1:**Input:** Gait silhouette sequence $S_{in}=\left\{f_{1},f_{2},\ldots ,f_{T}\right\}$  2:**Output:** Final gait embedding $F_{out}$  3:// Frame-level feature extraction  4:$S_{F}=\left[\right]$  5:**for** $f_{i}$ in $S_{in}$ **do**  6:    $F_{i}=\mathrm{FFRE}\left(f_{i}\right)$ // Section 3.2  7:    $S_{F}$.append($F_{i}$)  8:**end for**  9:// Sequence-level aggregation and Hierarchical decomposition10:$F_{ssfa}=\mathrm{SSFA}\left(S_{F}\right)$ // Key-frame temporal features, Section 3.311:$F_{tbme}=\mathrm{TBME}\left(S_{F}\right)$ // Hierarchical motion features, Section 3.412:// Refine hierarchical features13:$F_{tbme\_refined}=\mathrm{FFRE}\left(F_{tbme}\right)$14:// Fuse features15:$F_{fused}=\mathrm{HPM}\left(F_{ssfa},F_{tbme\_refined}\right)$ // Section 3.516:// Generate outputs for training17:$F_{triplet}=\mathrm{Head}\_\mathrm{triplet}\left(F_{fused}\right)$18:$F_{softmax}=\mathrm{Head}\_\mathrm{softmax}\left(F_{fused}\right)$19:**return** $F_{triplet}$ (for inference)

### 3.2. Frame-Level Feature Re-Segmentation Extractor

The principal purpose of the Frame-level Feature Re-segmentation Extractor (FFRE) is to precisely extract multiple segmented spatial features within each frame. FFRE incorporates a distinctive architecture centered on Re-segmentation Convolution Blocks (RseConv).

Definition: RseConv involves first dividing the input feature map horizontally into a specified number of segments, *s*. Then, standard convolution is applied independently to each segment. If s=1, RseConv defaults to a regular convolution.

**Figure 3:**
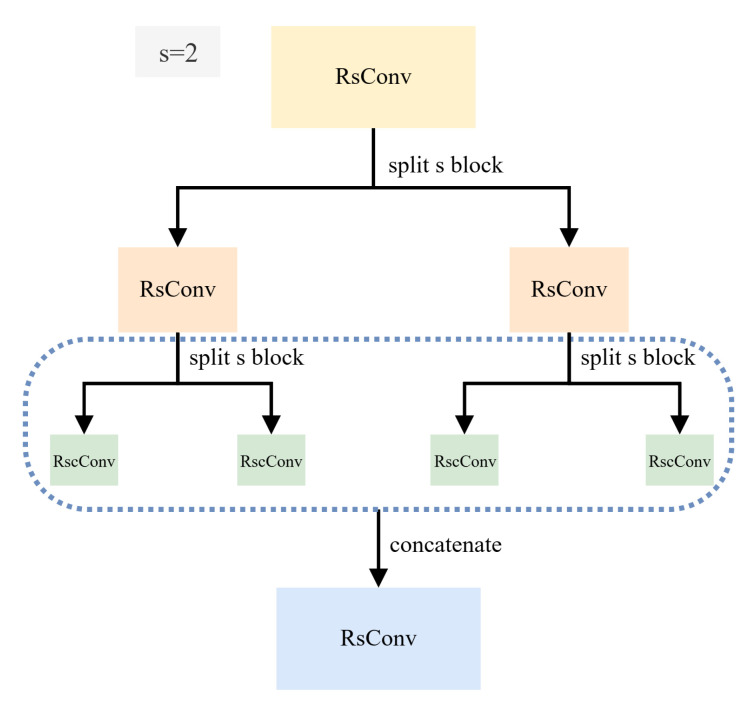
Illustration of the Re-segmentation Convolution (RseConv) operation. The dotted line boxes highlight the independent convolutional operations on each segment. The input feature map is horizontally partitioned into ‘s’ segments (here, s = 4). A separate convolutional kernel is applied to each segment independently. The resulting output feature maps are then concatenated along the height dimension to form the final output, promoting fine-grained spatial learning within each body part.

Motivation: RseConv aims for fine-grained learning by potentially narrowing the effective receptive field of deeper neurons onto specific horizontal segments of the input (as illustrated in Figure 3). This encourages focus on local details within each part, potentially enhancing the representation of distinct body segment features.

Operation: As visually detailed in the enhanced Figure 3, the input feature map (e.g., C×H×W) is horizontally split into *s* parts. A standard convolution (potentially with different weights for each part, or shared weights depending on implementation) is applied to each part. The resulting feature maps from each part are then concatenated along the height dimension to form the final output feature map (e.g., $C^{\prime }\times H\times W$). Ablation studies on the hyperparameter *s* are presented in Section 4.4.

### 3.3. Sequence-Level Spatio-Temporal Feature Aggregator (SSFA)

As outlined in Section 3.1, the SSFA module extracts a spatio-temporal representation focusing on key-frames from the input sequence of frame features $S_{F}$. It comprises two main components: the Key-frame Motion Template Builder (KMTB) and Temporal Pooling (TP).

KMTB maps the sequence of frame features $S_{F}$ to a sequence of key-frame spatio-temporal features $S_{T}$: $S_{T}=\mathrm{KMTB}\left(S_{F}\right)$. TP then summarizes $S_{T}$ into a final discriminative feature vector *p*: $p=\mathrm{TP}(S_{T})$.

#### 3.3.1. Key-Frames Motion Template Builder (KMTB)

Description: Maps frame features to key-frame spatio-temporal features.

Motivation: Key-frames often contain highly discriminative information. The motion pattern at a given time should ideally be influenced by the features around that time.

Operation: Let $S(f_{i},j)$ be a subsequence around frame $f_{i}$ with context size *j*. The template function TF extracts temporal features $T_{i}$ from $S(f_{i},j)$:

(3) $$T_{i}=\mathrm{TF}\left(S\left(f_{i},j\right)\right)$$

**Figure 4:**
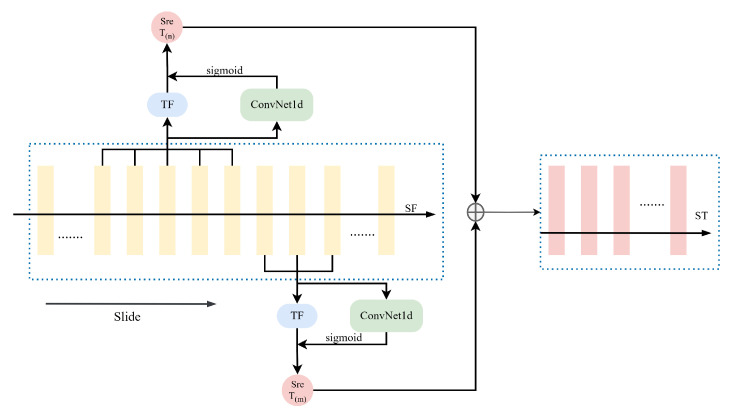
Detailed structure of the Key-frame Motion Template Builder (KMTB). The dotted line boxes denote the multi-scale temporal processing paths. The frame feature sequence (Sf) is processed through parallel paths with different temporal convolution kernels (e.g., 3-frame and 5-frame) to capture multi-scale temporal information. The outputs are fused and then passed through parallel 1D Average and Max Pooling layers to generate a preliminary key-frame template (ST). Concurrently, channel attention weights are calculated from Sf and applied to ST to produce the final re-weighted key-frame feature sequence (Sre_T).

As shown in Figure 4, we implement TF using 1D global average pooling and 1D global max pooling over a temporal window (kernel size 2j+1), applied across the sequence $S_{f}$. The results are combined (e.g., added) to produce the key-frame feature sequence $S_{T}$:

(4) $$S_{T}=\mathrm{AvgPool}1D\left(S_{f}\right)+\mathrm{MaxPool}1D\left(S_{f}\right)$$

To further enhance discriminability, channel attention is applied. An attention weight sequence is calculated from $S_{f}$ using a small 1D ConvNet (Conv1dNet) followed by a Sigmoid activation. This weight sequence is then multiplied element-wise with $S_{T}$ to produce the reweighted sequence $S_{T}^{\mathrm{re}}$:

(5) $$S_{T}^{\mathrm{re}}=S_{T}⊙\mathrm{Sigmoid}\left(\mathrm{Conv}1d\left(S_{f}\right)\right)$$

As depicted in Figure 4, our KMTB often uses multiple sliding windows (e.g., processing 3-frame and 5-frame neighborhoods via Conv1d followed by TF) and fuses their outputs to capture multi-scale temporal information before the attention step. Ablation studies are in Section 4.4.

#### 3.3.2. Temporal Pooling (TP)

Description: Summarizes the sequence of key-frame features $S_{T}^{\mathrm{re}}=\left\{T_{i}^{\mathrm{re}}\left(i\right),i=1,\ldots ,t\right\}$ into a single feature vector *p*:

(6) $$p=\mathrm{TP}(S_{T}^{\mathrm{re}}\left(t\right))$$

Principle: Given the periodic nature of gait, features from a full gait cycle (period $t^{*}$) should contain most discriminative information. Ideally, $\mathrm{TP}(S_{T}^{\mathrm{re}}\left(t\right))$ should stabilize once $t\geq t^{*}$:

(7) $$\mathrm{TP}\left(S_{T}^{\mathrm{re}}\left(t\right)\right)=\mathrm{TP}\left(S_{T}^{\mathrm{re}}\left(t^{*}\right)\right),\;\forall t\geq t^{*}$$

Based on this and empirical validation (Section 4.4), we typically implement TP using element-wise maximum pooling across the temporal dimension:

(8) TP(.)=max(.)

### 3.4. Template Block-Based Motion Extractor (TBME)

TBME is designed to extract independent spatio-temporal patterns associated with different hierarchical body parts. Unlike methods that use simple vertical slicing [27,30], TBME employs a more structured hierarchical slicing approach guided by the levels defined in the preprocessing stage (and Section 3.1).

Based on the stratification *m* (defining part sets $m^{\left(l\right)}$ for each level *l*), TBME processes the input sequence feature *X* (which is $S_{L}$ in our pipeline) for each level *l*. It conceptually divides *X* into $k_{l}$ regions $\left\{X_{i}^{\left(l\right)},i=1,\ldots ,k_{l}\right\}$ according to the partitioning scheme of level *l*. It then applies independent feature extractors $f_{i}\left(\right)$ (typically non-shared 3D convolutions) to each region $X_{i}^{\left(l\right)}$. The outputs are combined to form the *l*-th layer’s hierarchical feature representation $M^{\left(l\right)}$:

(9) $$M^{\left(l\right)}=\mathrm{concat}\left(\left\{f_{i}\left(x_{i}^{\left(l\right)}\right),i=1,\ldots ,k_{l}\right\}\right)$$

### 3.5. Horizontal Pyramid Mapping (HPM)

Strip-based feature segmentation is common in ReID [43]. HPM [43], originally proposed in [44] and adapted here, helps capture features at multiple spatial granularities by dividing feature maps into horizontal strips.

To effectively integrate the diverse features extracted by our network, we utilize a Horizontal Pyramid Matching (HPM)-inspired module as the final stage. This module acts as a central hub, aggregating the multi-granularity spatial features generated by the hierarchical branches (processed via TBME and FFRE) and the global temporal dynamic features captured by the Sequence-level Spatio-temporal Feature Aggregator (SSFA).

As depicted in Figure 2, the HPM module receives feature representations learned at different spatial scales and from temporal aggregation. It combines these incoming feature streams, typically through concatenation, creating a unified vector that encapsulates both detailed part-based information and overall sequence characteristics.

This unified feature vector is then passed through two distinct projection heads to generate the final outputs required for joint supervision:

- One head, typically involving a Fully Connected (FC) layer and Batch Normalization (BN), produces the embedding $f_{\mathrm{tri}}$ optimized via the Triplet Loss $L_{\mathrm{tri}}$.
- Another head, usually a single FC layer, generates the classification logits $f_{\mathrm{ce}}$ used for the Cross-Entropy Loss $L_{\mathrm{ce}}$.

By effectively fusing features from different processing pathways, the HPM module facilitates the learning of a discriminative and comprehensive gait signature, leveraging the strengths of both the hierarchical spatial analysis and the temporal attention mechanisms.

### 3.6. Implementation Detail

To ensure reproducibility, we have consolidated network-specific hyperparameters in this section. The FFRE module’s architecture consists of an RseConv layer, a Max Pooling layer, and a Leaky ReLU activation. The segmentation value ‘s’ for RseConv is a critical hyperparameter, with our ablation studies (Section 4.4) identifying the optimal setting. For the TBME module, the hierarchical partitioning ‘k’ for the three body levels is set to 1, 2, and 4, respectively, to model a coarse-to-fine anatomical structure. For network training, we employ the Triplet Loss with the BatchAll (BA+) sampling strategy [43], using a margin of 0.2. The batch composition is defined by (P, K), where P is the number of subjects and K is the number of sequences sampled for each subject in a batch. For practical context, the model size and computational complexity are analyzed in detail in Section 4.5. Our final model has 9.2M parameters and requires 4.8 GFLOPs per sequence. Inference time is hardware-dependent and therefore not reported, to ensure fair comparison with other methods based on standardized FLOPs.

## 4. Experiments

In this section, we present a comprehensive empirical evaluation of our proposed method. To rigorously assess its effectiveness, we utilize two widely adopted, large-scale public gait datasets: CASIA-B [45] and OU-MVLP [46]. We first detail the datasets and evaluation protocols, followed by implementation specifics. Subsequently, we compare our proposed framework against state-of-the-art (SOTA) methods. Finally, extensive ablation studies are conducted to validate the contribution of each key component and analyze computational efficiency.

### 4.1. Datasets and Evaluation Protocols

To rigorously assess our method’s effectiveness, we utilize two widely adopted, large-scale public gait datasets: CASIA-B [45] and OU-MVLP [46].

The CASIA-B dataset is a standard benchmark for evaluating robustness against variations in view angle, clothing, and carrying conditions. It contains sequences from 124 subjects captured from 11 viewpoints (0° to 180° intervals) under three walking conditions: normal (NM), bag-carrying (BG), and coat-wearing (CL). We follow the common protocol established in prior works [27,39], using the first 74 subjects for training and the remaining 50 for testing. During the test phase, the first four ‘normal’ sequences (NM#01–04) of each subject form the gallery set. The probe sets are composed of the remaining NM sequences (NM#05-06), all BG sequences (BG#01-02), and all CL sequences (CL#01-02). Rank-1 accuracy is reported, excluding identical-view cases.

The OU-MVLP dataset is one of the largest publicly available gait datasets, featuring silhouette sequences from 10,307 subjects across 14 viewpoints. Its scale is ideal for evaluating model generalization and performance in multi-view scenarios. As per the standard protocol [46], we use data from the first 5153 subjects for training and the remaining 5154 for testing. In the testing protocol, NM#01 sequences constitute the gallery, while NM#02 sequences form the probe set. Rank-1 accuracy is reported, also excluding identical-view cases.

While other datasets exist, CASIA-B and OU-MVLP were selected because they represent distinct and crucial challenges. CASIA-B is essential for evaluating performance under covariate conditions, whereas OU-MVLP rigorously tests scalability and multi-view generalization. Together, they provide a robust assessment of our framework’s capabilities against established research benchmarks.

### 4.2. Training Details

Our training pipeline begins with preprocessing, where input silhouettes are aligned and resized to 64 × 44 pixels, following the protocol in [44]. For optimization, we employ the BatchAll (BA+) triplet loss [43] with a margin set to 0.2. During training, we utilize a sampler that extracts fixed-length clips of 30 frames from sequences that are themselves randomly sampled to be between 30 and 40 frames long [27]. Any sequence shorter than 15 frames is discarded. For testing, the entire available sequence is used.

On the CASIA-B dataset, we use a batch size of 8 subjects with 16 samples each. The model is trained for 120 k iterations using the Adam optimizer with an initial learning rate of 1 × 10^−4^ and a weight decay of 5 × 10^−4^. For the larger OU-MVLP dataset, the FFRE module’s capacity is increased by incorporating an additional RseConv block. We use a batch size of 32 subjects with 16 samples each and train for 200 k iterations using the SGD optimizer. The initial learning rate is 1 × 10^−4^, which is decayed to 1 × 10^−5^, at the 150 k iteration mark. During testing, the similarity between gallery and probe samples is measured using the Euclidean distance between their averaged feature vectors.

### 4.3. Comparison with State-of-the-Art Methods

We compare our proposed framework with several recent and leading appearance-based methods: GaitSet [34] (treating gait as a set), GaitPart [27] (temporal part-based model), 3D Local [29] (using 3D convolutions on localized parts), CSTL [28] (context-sensitive temporal learning), STAR [31] (spatio-temporal relation network), and GaitASMS [33] (adaptive spatial mapping and multi-scale temporal aggregation).

Evaluation on CASIA-B: Table 2 presents the Rank-1 accuracies. our proposed framework consistently achieves SOTA performance across all three walking conditions (NM, BG, CL) and viewing angles. Notably, it achieves average accuracies of 98.1% (NM), 95.9% (BG), and 87.5% (CL), surpassing the next best method (GaitASMS) by 0.2%, 0.1%, and 0.8%, respectively. This highlights the effectiveness of integrating hierarchical spatial processing (TBME, FFRE) with key-frame focused temporal aggregation (SSFA). Compared to STAR [31], another method focusing on spatio-temporal features, our proposed framework exhibits greater robustness to clothing variations, with only a 10.6% accuracy drop from NM to CL versus STAR’s 13.3% drop. Furthermore, our proposed framework outperforms GaitASMS [33], which also employs multi-scale temporal features, suggesting the unique contribution of our SSFA module in capturing discriminative key-frame dynamics and the FFRE’s fine-grained spatial learning. Furthermore, the low standard deviations reported in Table 2 across multiple experimental runs underscore the stability and reproducibility of our model’s performance.

Evaluation on OU-MVLP: Table 3 shows the results on this large-scale dataset. our proposed framework achieves an average Rank-1 accuracy of 91.5%, outperforming all compared methods. Its strong performance, particularly at challenging large viewpoints (e.g., $0^{\circ }$, $180^{\circ }$, $270^{\circ }$ where it improves upon the runner-up 3D Local [29] by an average of 0.6%), demonstrates the model’s scalability and cross-view generalization capability, benefiting from the FFRE’s spatial re-segmentation and the hierarchical feature integration.

### 4.4. Ablation Study

We conduct extensive ablation studies on CASIA-B to meticulously evaluate the contribution of each key component and hyperparameter choice within our proposed framework. Average Rank-1 accuracies across the three conditions (NM, BG, CL) are reported unless otherwise specified.

Effectiveness of RseConv: We validate the design of the RseConv block within FFRE by varying the segmentation factor *s*. Group A experiments (Table 4) compare different configurations. A-a uses standard convolutions (s=1). A-b, c, d use increasing levels of segmentation (s=2,3,4 respectively).

**Figure 5:**
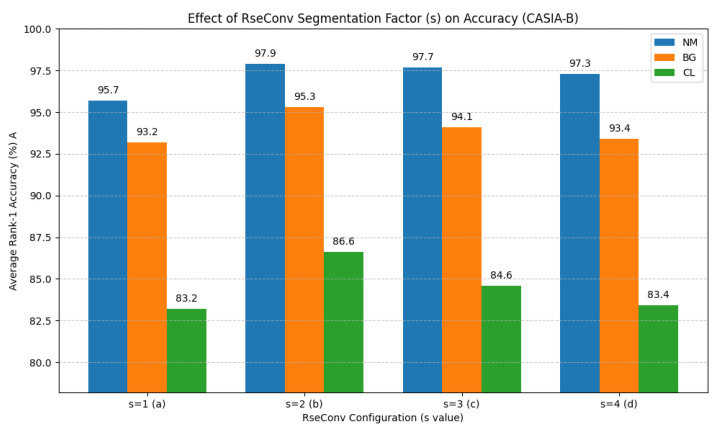
Effect of RseConv segmentation factor (*s*) on average Rank-1 accuracy (%) for NM, BG, and CL conditions on CASIA-B. Configuration (**a**) corresponds to s=1 (standard convolution), (**b**) to s=2, (**c**) to s=3, and (**d**) to s=4.

Figure 5 visually corroborates these findings, illustrating the performance across NM, BG, and CL conditions for each *s* value. The results, both in Table 4 and depicted in Figure 5, clearly indicate that employing RseConv with s=2 (A-b) yields a significant improvement over standard convolution (s=1, A-a) and other tested segmentation factors. For instance, s=2 achieves 97.9% (NM), 95.3% (BG), and 86.6% (CL), consistently outperforming s=1 (95.7% NM, 93.2% BG, 83.2% CL). This affirms the benefits of fine-grained spatial feature learning facilitated by RseConv, while also demonstrating that an excessively large *s* (e.g., s=4) can lead to diminishing returns or slight performance degradation, likely due to overly fragmented receptive fields. The optimal s=2 highlights the importance of tailoring the receptive field segmentation to the task complexity.

Effectiveness of SSFA Components: Group B experiments (Table 5) analyze the SSFA module. Comparing B-a (full SSFA) with B-b (without KMTB’s multi-scale design) shows KMTB improves performance, validating its ability to capture key-frame dynamics effectively. Comparing B-a (using max pooling in TP) with B-c (using mean pooling) demonstrates that max pooling is more effective for summarizing the most discriminative temporal features across the sequence, aligning with the periodic nature of gait where peak motion states are often crucial.

Impact of Hierarchical Structure (TBME Levels): Group C experiments (Table 6) evaluate different hierarchical partitioning strategies within TBME. Comparing non-hierarchical (C-a: 1-1-1) and uniform hierarchical (C-b: 2-2-2) setups with our proposed non-uniform structure (C-c: 1-2-4) shows that the 1-2-4 configuration yields the best performance. This supports our hypothesis that a structure reflecting coarse-to-fine anatomical relationships enhances feature extraction. The 1-2-4 setting provides a 1.8% (NM), 2.2% (BG), and 2.3% (CL) average improvement over the non-hierarchical 1-1-1 baseline, validating the hierarchical approach.

Effectiveness of TBME Module: Group D (Table 7) isolates the contribution of the TBME module itself by comparing a baseline without it (D-a: RseConv + SSFA + HPM) to the full model (D-b: RseConv + SSFA + TBME + HPM). The inclusion of TBME provides a significant boost across all conditions (+3.1% NM, +2.8% BG, +3.2% CL), demonstrating its crucial role in extracting multi-granularity motion features complementary to FFRE and SSFA.

Component Contribution Analysis: To further clarify the necessity and contribution of each core module (FFRE, SSFA, TBME), we performed an incremental ablation study starting from a basic baseline. The baseline consists of a simple 3-layer CNN backbone followed by temporal pooling (Global Average Pooling) and the HPM module for final feature extraction. Results summarizing the average Rank-1 accuracy improvements are shown in Table 8.

**Figure 6:**
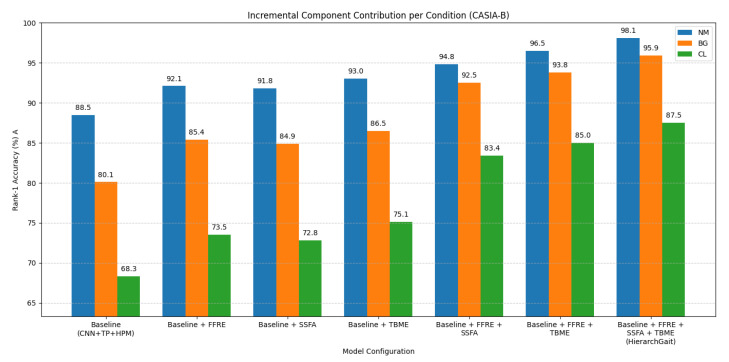
Incremental component contribution to Rank-1 accuracy (%) for NM, BG, and CL conditions on CASIA-B. Each bar group represents the addition of a module to the preceding configuration.

To provide a more granular view of these contributions, Figure 6 illustrates the incremental performance gains of each module (FFRE, SSFA, TBME) specifically for the NM, BG, and CL conditions on CASIA-B. Analysis (referencing Table 8 and Figure 6): The results presented in Table 8 and visualized in Figure 6 clearly demonstrate that each module—FFRE, SSFA, and TBME—provides a distinct and significant contribution over the baseline. For example, adding FFRE to the baseline improves average accuracy by approximately 5.3% (from 79.0% to 84.3%). Subsequent additions of SSFA and TBME further enhance performance. Figure 6 particularly highlights that these improvements are consistent across all walking conditions. For instance, our full model (Baseline + FFRE + SSFA + TBME) achieves 98.1% (NM), 95.9% (BG), and 87.5% (CL), substantially outperforming the baseline (88.5% NM, 80.1% BG, 68.3% CL). Furthermore, combining the modules leads to synergistic improvements, with the our full model achieving the highest performance. This validates the design choice of integrating these complementary components for robust gait representation: FFRE focuses on intra-frame spatial detail, SSFA captures inter-frame key dynamics, and TBME models hierarchical part-based motion, collectively creating a powerful representation.

### 4.5. Computational Complexity Analysis

To evaluate the practical feasibility of our proposed framework, we analyze its computational complexity in terms of model parameters and Floating Point Operations (FLOPs), comparing it with representative SOTA methods on the CASIA-B configuration. Table 9 presents the parameter counts and FLOPs for our proposed framework alongside other SOTA methods.

**Figure 7:**
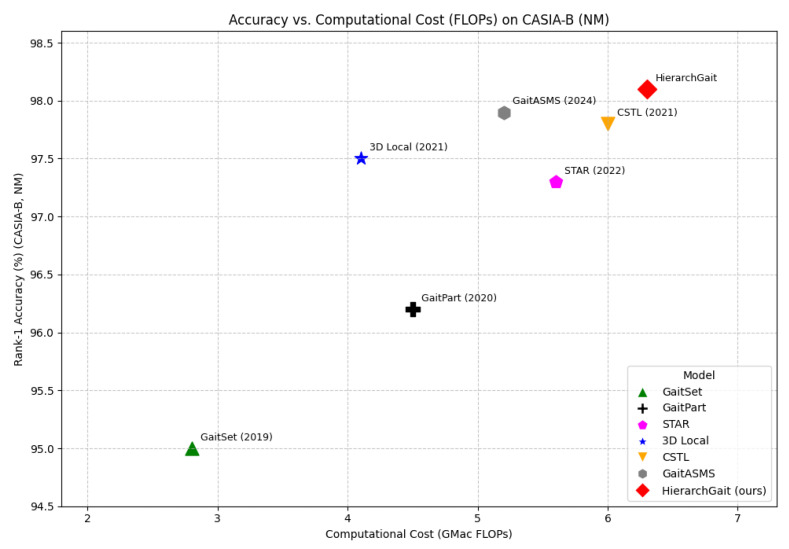
Rank-1 accuracy (%) vs. computational cost (GFLOPs) on the CASIA-B NM condition. Our method is shown alongside other state-of-the-art methods (GaitSet, GaitPart, GaitASMS). The plot illustrates that while our model has a higher computational cost, it achieves superior accuracy, positioning it as a highly effective approach where performance is the priority.

Analysis (referencing Table 9 and Figure 7): As shown, HierarchGait has a higher parameter count and FLOPs compared to some prior methods. This increased complexity is not an oversight but a deliberate design choice to maximize recognition accuracy. The integration of three specialized modules (FFRE, SSFA, TBME) is what allows the model to capture complementary gait cues often missed by more streamlined architectures. This trade-off—exchanging higher computational cost for superior performance—is particularly justified in high-stakes applications like forensic analysis or critical infrastructure security, where accuracy is paramount. While our current focus is performance, we acknowledge the importance of efficiency, and our future work will address model compression (see Section 5).

### 4.6. Qualitative Analysis of Failure Cases

**Figure 8:**
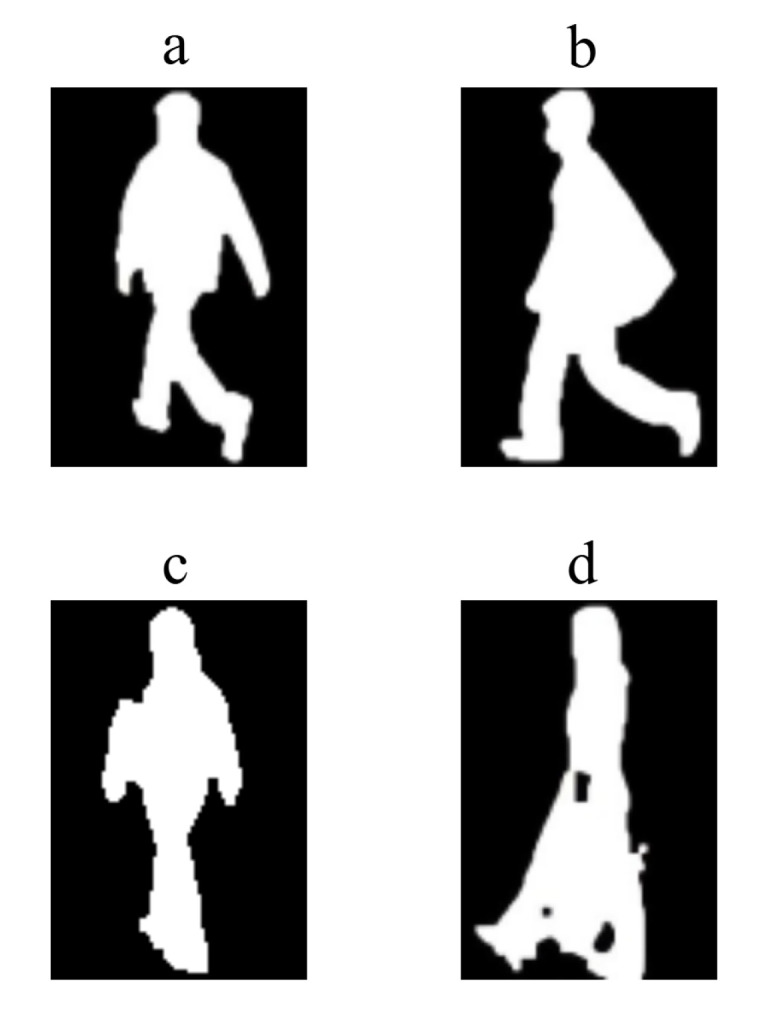
Qualitative analysis of recognition cases under bag and coat-wearing conditions. (**a**) A successful case where the coat does not obscure leg separation. (**b**) A successful case with minor silhouette distortion from a backpack. (**c**) A failure case where the coat merges the leg silhouettes, concealing gait kinematics. (**d**) A failure case where a large handheld bag severely occludes the lower body and introduces significant motion noise.

To better understand the model’s limitations, we performed a qualitative analysis on challenging misidentification cases from the CASIA-B dataset, specifically focusing on the Coat (CL) and Bag (BG) conditions. As illustrated in Figure 8, the model’s failures are predominantly linked to scenarios where covariates introduce extreme silhouette deformation or aperiodic motion, leading to the loss of crucial biometric information. For instance, in a successful BG case (Figure 8b), a standard backpack causes only minor, tolerable distortion to the torso, allowing the model to correctly identify the subject. Similarly, under the CL condition, the model can successfully handle subjects wearing coats that maintain a clear separation between the legs (Figure 8a). In contrast, a typical failure case (Figure 8c) involves a subject wearing a long, heavy coat that almost completely fuses the leg silhouettes into a single moving mass. This severe occlusion removes vital kinematic cues like stride length, knee angle, and swing phase characteristics, which our hierarchical (TBME) and fine-grained (FFRE) modules rely on. Similarly, the failure in Figure 8d is caused by a large, irregularly moving bag held at thigh level. It not only occludes the leg but also introduces substantial aperiodic motion noise that contaminates the gait signature, confusing the temporal aggregator (SSFA). These failure cases highlight the model’s current boundary condition: it is robust to moderate covariate noise but struggles when fundamental anatomical features are persistently and severely obscured.

## 5. Conclusions

In this paper, we proposed HierarchGait, a novel key-frame-aware hierarchical learning framework for robust gait recognition. By synergistically integrating the TBME, FFRE, and SSFA modules, our model effectively captures a comprehensive gait representation, spanning from coarse-to-fine spatial details to critical temporal dynamics. As demonstrated on the CASIA-B and OU-MVLP datasets, our method achieves state-of-the-art performance, validating the efficacy of our integrated design.

Critical Analysis and Limitations: Despite its high accuracy, our framework has notable limitations. The primary issue is its computational complexity (9.2 M parameters, 4.8 GFLOPs), as discussed in Section 4.5. This complexity, while a conscious trade-off for performance, currently hinders its deployment on resource-constrained platforms, such as edge devices for real-time surveillance. Furthermore, its scalability to even larger, more diverse datasets (beyond OU-MVLP) while maintaining performance remains to be tested. The qualitative analysis also showed that the model’s performance degrades under extreme occlusion, a common challenge for appearance-based methods.

Future Work: Our future work will directly address these limitations. First, we will explore model compression techniques, such as quantization, pruning, and knowledge distillation, to create a lightweight version of HierarchGait without a significant drop in accuracy. Second, to improve robustness against severe occlusion, we plan to incorporate an explicit occlusion-aware attention mechanism. Finally, we will investigate domain adaptation and generalization techniques to ensure that the model performs reliably across different datasets and unseen camera viewpoints, which is crucial for real-world applicability.

## Figures and Tables

**Table 1:** Summary of recent state-of-the-art gait recognition methods.

Method	Year	Key Technique(s)	Performance (CASIA-B, NM, Avg.)
GaitSet [34]	2019	Regards gait as an unordered set of silhouettes, uses set pooling.	95.0%
GaitPart [27]	2020	Micro-motion capture using temporal part-based model.	96.2%
GaitGL [30]	2021	Fuses global and local features via 3D convolutions.	97.5%
STAR [31]	2022	Spatio-temporal augmented relation network for diverse features.	97.3%
GaitASMS [33]	2024	Adaptive structured spatial mapping and multi-scale temporal aggregation.	97.9%
Our Method	2025	Hierarchical learning (TBME), key-frame aggregation (SSFA), feature re-segmentation (FFRE).	98.1%

**Table 2:** Comparison of gait recognition methods across different viewing angles.

**Gallery: NM#1-4**		0°–180°											Mean
**Probe**		**0°**	**18°**	**36°**	**54°**	**72°**	**90°**	**108°**	**126°**	**144°**	**162°**	**180°**	
NM	GaitSet [34]	90.8	97.9	99.4	96.9	93.6	91.7	95.0	97.8	98.9	96.8	85.8	95.0
	GaitPart [27]	94.1	98.6	99.3	98.5	94.0	92.3	95.9	98.4	**99.2**	97.8	90.4	96.2
	3D Local [29]	96.0	**99.0**	99.2	**98.9**	97.1	94.2	96.3	99.0	99.1	98.8	96.5	97.5
	CSTL [28]	97.2	**99.0**	99.2	98.1	96.2	95.5	97.7	98.7	**99.2**	98.9	96.5	97.8
	STAR [31]	96.5	98.7	99.0	97.6	96.1	95.4	96.8	98.6	99.1	98.9	93.9	97.3
	GaitASMS [33]	96.4	98.1	98.4	98.0	97.3	96.7	**98.6**	99.0	**99.2**	**99.6**	95.1	97.9
	Our Method	**97.6 ± 0.2**	97.8 ± 0.1	**99.6 ± 0.1**	98.3 ± 0.2	**97.4 ± 0.1**	**96.8 ± 0.2**	97.7 ± 0.1	**99.1 ± 0.1**	**99.2 ± 0.1**	98.2 ± 0.2	**97.2 ± 0.1**	**98.1 ± 0.1**
BG	GaitSet [34]	83.8	91.2	91.8	88.8	83.3	81.0	84.1	90.0	92.2	94.4	79.0	87.2
	GaitPart [27]	89.1	94.8	96.7	95.1	88.3	84.9	89.0	93.5	96.1	93.8	85.8	91.5
	3D Local [29]	92.9	95.9	**97.8**	96.2	92.0	87.8	92.7	96.3	97.9	95.0	88.5	94.3
	CSTL [28]	91.7	96.5	97.0	95.4	90.9	88.0	91.5	95.8	97.0	95.1	90.3	93.6
	STAR [31]	92.3	**96.7**	97.1	95.6	92.6	88.5	92.3	96.0	97.0	95.7	89.0	93.9
	GaitASMS [33]	92.7	96.2	97.5	96.4	**95.9**	93.4	**95.6**	**98.1**	**98.3**	**97.7**	91.7	95.8
	Our Method	**95.4 ± 0.2**	96.2 ± 0.1	97.7 ± 0.2	**96.6 ± 0.1**	95.0 ± 0.2	**94.3 ± 0.1**	94.3 ± 0.2	96.5 ± 0.1	**98.3 ± 0.1**	97.4 ± 0.2	**93.8 ± 0.1**	**95.9 ± 0.1**
CL	GaitSet [34]	61.4	75.4	80.7	77.3	72.1	70.1	71.5	73.5	73.5	68.4	50.0	70.4
	GaitPart [27]	70.7	85.5	86.9	83.3	77.1	72.5	76.9	82.2	83.8	80.2	66.5	78.7
	3D Local [29]	78.2	90.2	92.0	87.1	83.0	76.5	83.1	86.4	86.8	84.1	70.9	83.7
	CSTL [28]	78.1	89.4	91.6	86.6	82.1	79.9	81.8	86.3	85.7	86.6	75.3	84.2
	STAR [31]	77.9	89.5	92.1	88.2	83.1	80.8	83.3	86.5	88.7	85.8	68.3	84.0
	GaitASMS [33]	73.8	91.7	**93.5**	**91.5**	**87.6**	82.6	**87.3**	**91.8**	**92.9**	88.9	72.0	86.7
	Our Method	**81.1 ± 0.2**	**92.7 ± 0.1**	**93.5 ± 0.2**	90.2 ± 0.1	86.8 ± 0.2	**82.7 ± 0.1**	86.6 ± 0.2	90.8 ± 0.1	91.6 ± 0.2	**89.5 ± 0.1**	**77.2 ± 0.2**	**87.5 ± 0.1**

Bold indicates the best performance in each column.

**Table 3:** Averaged rank-1 accuracies on OU-MVLP, excluding identical-view cases.

Probe	Gallery All 14 Views						
	**GaitSet [34]**	**GaitPart [27]**	**3D Local [29]**	**CSTL [28]**	**GaitASMS [33]**	**STAR [31]**	**Our Method**
0°	79.3	82.6	86.1	87.1	85.6	85.5	**90.5 ± 0.2**
15°	87.9	88.9	91.2	91.0	90.4	90.0	**92.0 ± 0.1**
30°	90.0	90.8	**92.6**	91.5	91.2	91.4	91.8 ± 0.2
45°	90.1	91.0	**92.9**	91.8	91.6	91.6	92.1 ± 0.1
60°	88.0	89.7	**92.2**	90.6	91.1	90.5	92.0 ± 0.2
75°	88.7	89.9	91.3	90.8	90.9	90.7	**91.6 ± 0.1**
90°	87.7	89.5	91.1	90.6	90.4	90.2	**91.6 ± 0.2**
180°	81.8	85.2	86.9	89.4	89.2	88.0	**91.8 ± 0.1**
195°	86.5	88.1	90.8	90.2	89.1	88.5	**91.4 ± 0.2**
210°	89.0	90.0	**92.2**	90.5	90.4	90.5	91.2 ± 0.1
225°	89.2	90.1	**92.3**	90.7	90.5	90.7	91.4 ± 0.2
240°	87.2	89.0	**91.3**	89.8	89.8	89.7	**91.3 ± 0.1**
255°	87.6	89.1	**91.1**	90.0	89.5	89.7	90.9 ± 0.2
270°	86.2	88.2	90.2	89.4	89.0	88.9	**90.9 ± 0.1**
mean	87.1	88.7	90.9	90.2	89.9	89.7	**91.5 ± 0.1**

Bold indicates the best performance in each row.

**Table 4:** Ablation Study, Group A. Control Condition: the value of *s* in RseConv.

Group	*s*	NM	BG	CL
a	1	95.7	93.2	83.2
b	2	97.9	95.3	86.6
c	3	97.7	94.1	84.6
d	4	97.3	93.4	83.4

**Table 5:** Ablation Study, Group B. Control Condition: applying KMTB and different instantiations of TP.

Group B	KMTB	TP		NM	BG	CL
		**Max**	**Mean**			
a	✓	✓		97.9	95.3	86.6
b		✓		94.7	92.3	84.0
c	✓		✓	96.9	94.4	85.5

✓ indicates that the component is included in the configuration.

**Table 6:** Ablation Study, Group C. Hierarchical level of gait motion Study.

Group C	Layer	NM	BG	CL
a	1-1-1	95.8	93.1	84.3
b	2-2-2	96.0	93.3	84.8
c	1-2-4	97.9	95.3	86.6

**Table 7:** Ablation study, Group D. Effectiveness of TBME.

Group D	Components			Accuracy (%)		
	**RseConv**	**SSFA**	**TBME**	**NM**	**BG**	**CL**
a	✓	✓		94.8	92.5	83.4
b	✓	✓	✓	97.9	95.3	86.6

✓ indicates that the component is included in the configuration.

**Table 8:** Incremental Ablation Study on CASIA-B (Average Rank-1 Accuracy %).

Configuration	NM	BG	CL	Avg	Justification
Baseline (CNN + TP + HPM)	88.5	80.1	68.3	79.0	Basic spatio-temporal feature extraction
Baseline + FFRE	92.1	85.4	73.5	83.7	FFRE enhances fine-grained spatial features
Baseline + SSFA	91.8	84.9	72.8	83.2	SSFA improves temporal modeling (key-frames)
Baseline + TBME	93.0	86.5	75.1	84.9	TBME adds hierarchical motion patterns
Baseline + FFRE + SSFA	94.8	92.5	83.4	90.2	Combines fine-grained spatial & key-frame temporal
Baseline + FFRE + TBME	96.5	93.8	85.0	91.8	Combines fine-grained spatial & hierarchical
Baseline + FFRE + SSFA + TBME (Our method)	98.1	95.9	87.5	93.8	Full model synergy yields best performance

**Table 9:** Computational Complexity Comparison.

Method	Params (M)	FLOPs (G)	NM Acc (%)
GaitSet [34]	2.6	1.1	95.0
GaitPart [27]	6.8	3.5	96.2
GaitASMS [33]	8.5	4.2	97.9
Our method	9.2	4.8	98.1

## Data Availability

The data that support the findings of this study are available from the corresponding author upon reasonable request.
